# Genetic diversity of *Plasmodium falciparum* in Grande Comore Island

**DOI:** 10.1186/s12936-020-03384-5

**Published:** 2020-09-03

**Authors:** Nasserdine Papa Mze, Hervé Bogreau, Cyrille K. Diedhiou, Vendela Herdell, Silai Rahamatou, Amy K. Bei, Sarah K. Volkman, Leonardo Basco, Souleymane Mboup, Ambroise D. Ahouidi

**Affiliations:** 1 Laboratory of Bacteriology-Virology, Hospital Aristide Le Dantec, BP 7325 Dakar, Senegal; 2 Institut de Recherche en Santé, de Surveillance Épidémiologique et de Formations, Arrondissement 4 Rue 2D1 Pôle Urbain de Diamniadio, Dakar, Senegal; 3 Aix Marseille Univ, IRD, AP-HM, SSA, VITROME, Marseille, France; 4 IHU-Méditerranée Infection, Marseille, France; 5 Laboratory of National Malaria Control Program, Moroni, Comoros; 6 Département Microbiologie et Maladies Infectieuses, Institut de Recherche Biomédicale des Armées, Unité Parasitologie et Entomologie, Marseille, France; 7 Centre National de Référence du Paludisme, Marseille, France; 8 Karolinska Institutet, Berzelius väg 3, 17177 Stockholm, Sweden; 9 Department of Epidemiology of Microbial Diseases, Yale School of Public Health, New Haven, CT 06510 USA; 10 Broad Institute: The Broad Institute of MIT and Harvard, Cambridge, MA 02142 USA

**Keywords:** Malaria, SNPs, *msp1*, *msp2*, Comoros

## Abstract

**Background:**

Despite several control interventions resulting in a considerable decrease in malaria prevalence in the Union of the Comoros, the disease remains a public health problem with high transmission in Grande Comore compared to neighbouring islands. In this country, only a few studies investigating the genetic diversity of *Plasmodium falciparum* have been performed so far. For this reason, this study aims to examine the genetic diversity of *P. falciparum* by studying samples collected in Grande Comore in 2012 and 2013, using merozoite surface protein 1 (*msp1*), merozoite surface protein 2 (*msp2*) and single nucleotide polymorphism (SNP) genetic markers.

**Methods:**

A total of 162 positive rapid diagnostic test (RDT) samples from Grande Comore were used to extract parasite DNA. Allelic families K1, Mad20 and RO33 of the *msp1* gene as well as allelic families IC3D7 and FC37 of the *msp2* gene were determined by using nested PCR. Additionally, 50 out of 151 samples were genotyped to study 24 SNPs by using high resolution melting (HRM).

**Results:**

Two allelic families were predominant, the K1 family of *msp1* gene (55%) and the FC27 family of *msp2* gene (47.4%). Among 50 samples genotyped for 24 SNPs, 42 (84%) yielded interpretable results. Out of these isolates, 36 (85%) were genetically unique and 6 (15%) grouped into two clusters. The genetic diversity of *P. falciparum* calculated from *msp1* and *msp*2 genes and SNPs was 0.82 and 0.61, respectively.

**Conclusion:**

In summary, a large genetic diversity of *P. falciparum* was observed in Grande Comore. This may favour persistence of malaria and might be one of the reasons for the high malaria transmission compared to neighbouring islands. Further surveillance of *P. falciparum* isolates, mainly through environmental management and vector control, is warranted until complete elimination is attained.

## Background

Despite several interventions to prevent, control, and eliminate malaria, 219 million cases and 435,000 deaths occurred worldwide in 2018, mainly in children under 5 years and pregnant women in Africa [1]. *Plasmodium falciparum* remains the most dominant species in Africa and responsible for the most severe clinical forms; it is associated with 95% of malaria-associated deaths [2, 3]. Many factors contribute to the severity of *P. falciparum* infections, such as the level of acquired immunity of the individual and genetic characteristics of the parasite [4].

The genetic diversity of *P. falciparum* remains an obstacle to achieve malaria elimination. Previous vaccine trials have shown limited efficacy of interventions in time and different geographic regions due to genetic polymorphisms [5, 6]. The genes coding for merozoite surface protein-1 (MSP1) and merozoite surface protein-2 (MSP2) have been widely used as genetic markers to determine allelic polymorphisms of *P. falciparum* [7–9]. The genome of *P. falciparum* is characterized by substitutions of single nucleotides occurring at specific positions in the genome, referred to as single nucleotide polymorphisms (SNPs). Approximately 112,000 SNPs have been described in the genome of *P. falciparum* and are considered useful molecular markers for barcoding to identify and characterize genetic diversity of *P. falciparum* [10–12].

In the Comoros archipelago, which is composed of four islands in the Indian Ocean between Madagascar and the African coast, malaria remains a major public health problem with high transmission and mortality rate among children under five years old [2]. Since 2004, Comoros has dramatically increased deployment of intervention strategies, including insecticide-treated bed nets, the treatment of patients with artemisinin-based combination therapy and the introduction of intermittent preventive treatment (IPT) in pregnant women. These strategies have resulted in a significant decrease in malaria infection, from 54,078 cases in 2004 to 1,072 in 2015 [13]. However, extensive interventions against malaria might as well result in changes in the population structure of *Plasmodium* species. First, because high genetic diversity has been associated with high levels of malaria transmission [14], a measure of genetic diversity can provide an indirect assessment of the effectiveness of an intervention [15]. Second, a high genetic structure of parasite populations, observable when gene flows between populations remain limited, allows for localized interventions. Conversely, the absence of population structure imposes the need for larger-scale strategies to be effective in the long term. Consequently, an estimation of genetic diversity in local *P. falciparum* isolates can provide important information on how to improve strategies to reach malaria elimination.

A 2010 study revealed a significant diversity of *P. falciparum* strains in the Union of Comoros where the mean expected heterozygosity (He) was 0.63 in Grande Comore and the multiplicity of infection (MOI) was 1.39 from microsatellite data [16]. Since this study, only one study has been conducted to investigate genetic diversity of the parasite in Comoros [17]. The results of this latter study showed a decrease in genetic diversity and in MOI based on *msp* genes correlated with a decrease in malaria transmission in Grande Comore.

As a follow-up of that study, the aim of the present study was to characterize *P. falciparum* populations in other sites in Grande Comore using *msp1*, *msp2* and SNPs to determine the genetic diversity and MOI.

## Methods

### Study site

**Fig. 1:**
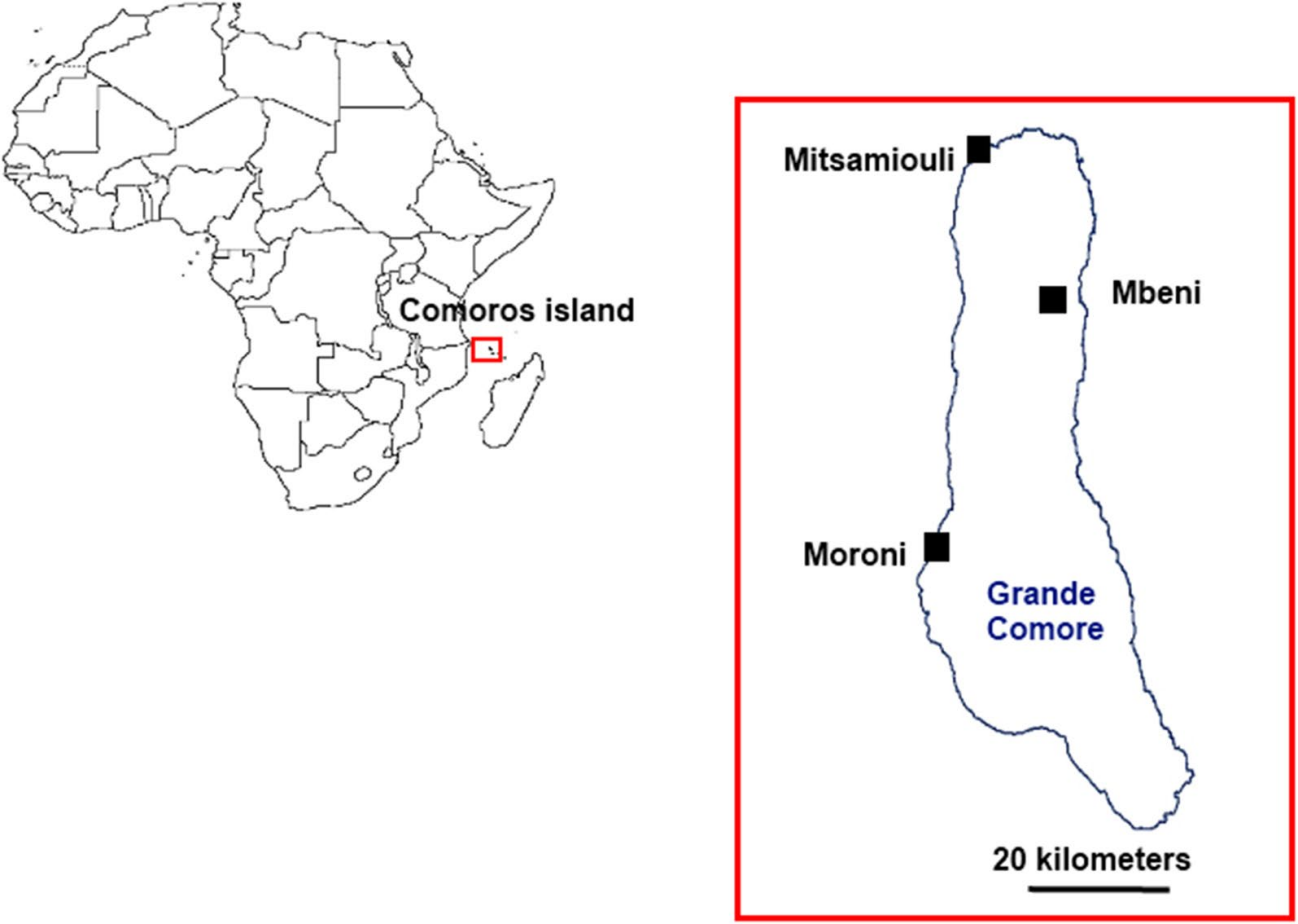
Map of Grande Comore Island showing the study sites

A total of 162 rapid diagnostic tests (RDTs) were collected in 2012 and 2013 in three sites of Grande Comore (Moroni n = 26, Mitsamiouli n = 59, and Mbeni n = 77). Malaria transmission varies in these three sites: hypoendemic in Moroni, mesoendemic in Mitsamiouli, and hyperendemic in Mbeni [16] (Fig. 1). The following RDTs were used in Grande Comore at the time of the present study: CareStart™ Malaria HRP2/pLDH Combo RDT (Access Bio, Inc., Somerset, NJ) and Standard Diagnostics Bioline Malaria Ag Pf/Pan test (SD Bioline/Abbott, Yongin-si, Gyeonggi-do, Republic of Korea). RDTs that were positive for *P. falciparum* were collected from health centres. RDTs that were negative or invalid were excluded from this study. All RDTs were stored at room temperature until they were used for DNA extraction.

### DNA extraction

Parasite DNA stored on the nitrocellulose membrane of RDTs was extracted according to the protocol developed by Cnops et al*.* [18]. DNA extraction was performed using QIAmp DNA or Blood Mini kit (Qiagen, Germantown, MD) following the manufacturer’s instructions.

### Genotyping of *msp1* and *msp2*

*Msp1* and *msp2* genes were amplified by nested PCR as described in earlier studies [19, 20]. All PCR amplifications were carried out in a total volume of 20 μl containing 6 μl Gotaq^®^ DNA polymerase-reaction buffer (Promega Corporation; Madison, WI), 0.5 μM of each primer, and 11 μl reagent grade water. The cycling conditions for the primary PCR were as follows: initial denaturation at 95 °C for 5 min, followed by 35 cycles of 94 °C for 1 min, 58 °C for 2 min, 72 °C for 2 min, with a final extension cycle of 72 °C for 3 min. The cycling conditions for the nested PCR were as follows: initial denaturation at 95 °C for 5 min, followed by 35 cycles of 94 °C for 1 min, 61 °C for 2 min, 72 °C for 2 min, with a final extension cycle of 72 °C for 3 min. Positive controls and negative control were run in each PCR. PCR products were analysed by gel electrophoresis on 2% agarose gels and visualized under ultraviolet trans-illumination (Biorad Gel Doc™ XR + System with Image Lab). A 100 bp DNA ladder (Quick-Load) was used to determine the size of PCR products.

The expected sizes of PCR products for the *msp1* gene were between 150–300 bp, 130–230 bp, and 150–200 bp for K1, MAD, and RO33, respectively. For the *msp2* gene, the expected sizes were between 290–450 bp for FC27 and between 450–700 bp for IC3D7.

### Genotyping of SNPs

Twenty-four SNPs of *P. falciparum* were genotyped by using high-resolution melting (HRM), as previously described [21]. PCR amplifications were performed using 2.5 × Light Scanner master mix (Biofire Diagnostics, Inc., Salt Lake City, UT), with forward primers at a final concentration of 0.05 μM, reverse primers at a final concentration of 0.2 μM, and allele-specific probes at a final concentration of 0.2 μM, and 1 μl of genomic DNA. SNPs were identified with a high minor allele frequency. The cycling and melting conditions for SNPs were as follows: 95 °C denaturation for 2 min followed by 55 cycles of 95 °C for 5 s and 56 °C for 30 s, then a pre-fusion cycle of 5 s each at 95 °C and 37 °C, followed by a melt from 35 °C to 90 °C at 0.30 °C/s.

### Statistical analysis

Genetic diversity of *msp1* and *msp2* genes of the 3 populations (Moroni, Mitsamiouli, and Mbeni) was determined by the number of alleles per locus and by Nei [22] unbiased expected heterozygosity index (He) using GENETIX software version 4.05 [23].

$$He=\left(\frac{2n-1}{2n}\right)\left(1-\sum_{i}{x}_{i}^{2}\right)$$

where n is the sample size and ${x}_{i}$ is the frequency of _i_ allele.

Genetic diversity index based on SNPs, equivalent to the expected heterozygosity index (He), was estimated by $\pi$ diversity index [24], implemented with MEGA X version 10.1 [25]. Phylogenetic tree, based on SNPs data, was constructed using MEGA X unweighted pair group method with arithmetic mean (UPGMA) algorithm [26].

Fisher’s exact test was used to compare the allelic frequencies of *msp1* and *msp2* genes in three regions of the Grande Comore and He. The MOI is the mean number of fragments detected for each *msp* gene and each allelic family. A sample with one clone for the *msp* genes was considered monoclonal, while a sample with at least 2 clones was considered polyclonal. Mann–Whitney U test was used to compare mean MOI. The significance threshold was fixed at α = 0.05.

## Results

### Genotyping of *msp1* and *msp2*

Of 162 samples, 151 were successfully genotyped: 25 from Moroni, 49 from Mitsamiouli, and 77 from Mbeni. Among them, 21 individual alleles were found, including 12 alleles for *msp1* gene and 9 alleles for *msp2* gene.

**Fig. 2:**
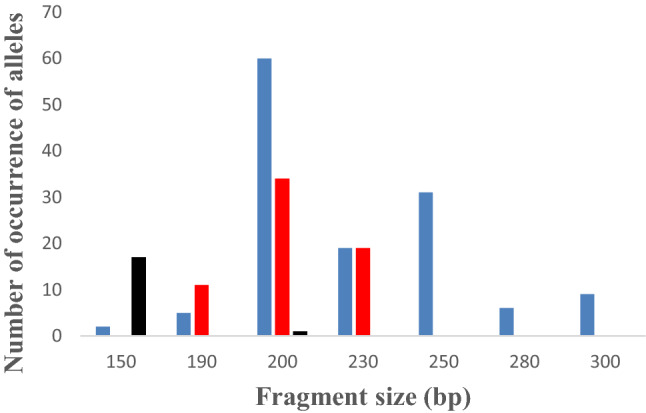
The numbers of occurrence of alleles for each *msp1* allelic family K1 (blue), MAD20 (red), and RO33 (black) are shown in the figure. The total numbers of K1, MAD20, and RO33 alleles are 134, 64 and 18, respectively

**Fig. 3:**
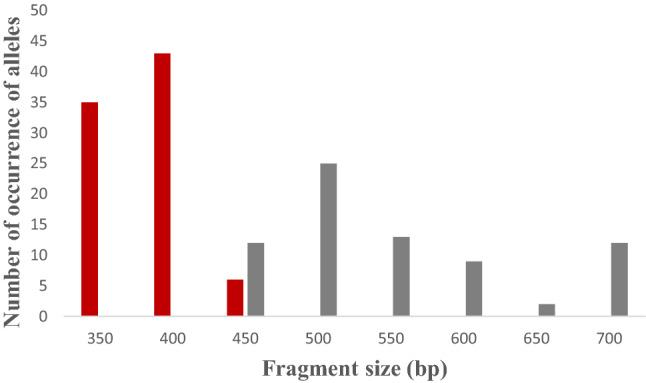
The numbers of occurrence of alleles for each *msp2* allelic family FC27 (red) and IC3D7 (gray) are shown in the figure. The total numbers of FC27 and IC3D7 alleles are 84 and 73, respectively

For *msp1*, 7 K1 type alleles (150–300 bp), 3 MAD20 type alleles (190–230 bp), and 2 RO33 type alleles (150 and 200 bp) were observed (Fig. 2). The most frequent K1, RO33, and MAD20 type alleles were 200 bp (60/132), 150 bp (17/18), and 200 bp (34/64) fragments, respectively. For *msp2*, 3 FC27 type alleles (350–450 bp) and 6 IC3D7 type alleles (450–700 bp) were obtained. For FC27, the most frequent type allele was 400 bp fragment, and the most frequent type allele for IC3D7 was 500 bp fragment (Fig. 3).

**Fig. 4:**
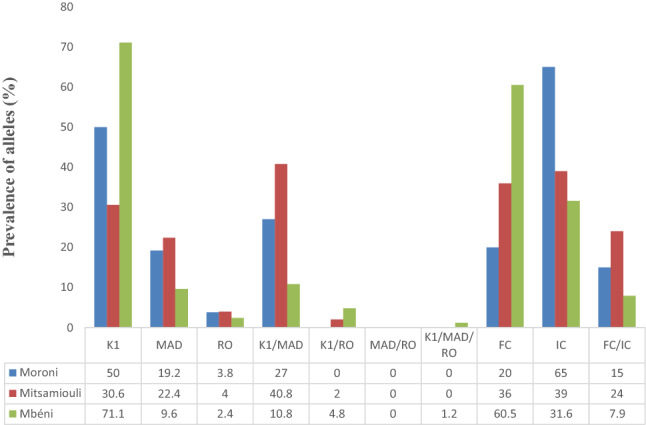
Allelic frequencies of *msp1* and *msp2* genes in 3 sites of Grande Comore

In all three sites, the K1 family was predominant (55%) for *msp1* gene (Table 1). It was more frequent in Mbeni (71.1%) compared to Moroni (50%; p = 0.059) and Mitsamiouli (30.6%; p < 0.01) (Fig. 4). There was no significant difference of K1 frequency between Moroni and Mitsamiouli (p > 0.05). However, MAD20 *msp1* allelic family was more frequently observed in Mitsamiouli (22.4%) than in Moroni (19.2%; p = 1) or Mbeni (9.6%; p = 0.29). RO33 *msp1* allelic family was poorly represented (2.4–4.0%) in all three sites.

**Table 1:** Genetic diversity of *P. falciparum msp* genes in Grande Comore

Gene	Allelic family	n (%)
*msp1*	K1	87 (55.0)
	MAD20	24 (15.2)
	RO33	5 (3.2)
	K1/MAD	36 (22.8)
	K1/RO33	5 (3.2)
	MAD/RO33	0 (0)
	K1/MAD20/RO33	1 (0.6)
	Total K1	129 (81.6)
	Total MAD20	61 (38.6)
	Total RO33	11 (7)
*msp2*	FC27	65 (47.4)
	IC3D7	53 (38.7)
	FC27/IC3D7	19 (13.8)
	Total FC27	84 (61.3)
	Total IC3D7	72 (52.5)

There was a total of 158 *msp1* and 137 *msp2* fragments

For *msp2* gene, IC3D7 allelic family was predominant in Moroni (65%). Its frequency was lower in both Mitsamiouli (39%; p = 0.80) and Mbeni (31.6%; p = 0.36). The FC27 family was found to be predominant in Mbeni (60.5%), in comparison to Moroni (20%; p < 0.01) and Mitsamiouli (36%; p = 0.019).

In Moroni, a total of 10 (40%) samples showed polyclonal infection with at least two clones, resulting in mean MOI of 1.40 **(**Table 2). In Mitsamiouli, 26 (53%) samples showed polyclonal infections with at least two clones (MOI = 1.57) and in Mbeni 26 (33.8%) samples were polyclonal (MOI = 1.35). There was no significant difference in MOI between Moroni and Mitsamiouli (p = 0.30). In Mbeni, 26 (33.8%) samples were polyclonal, and mean MOI was 1.35. The difference in MOI was not statistically significant between Mbeni and Mitsamiouli (p = 0.06) and between Moroni and Mbeni (p = 0.67).

**Table 2:** Multiplicity of infection (MOI) based on *msp1* and *msp2* genes in *P. falciparum* isolates from Moroni, Mitsamiouli, and Mbeni

Sites	Moroni	Mitsamiouli	Mbeni	All sites
*msp1*	1.46	1.46	1.29	1.36
*msp2*	1.00	1.06	1.06	1.05
*msp1* + *msp2*	1.40	1.57	1.35	1.43

The levels of endemicity were hypoendemic in Moroni (n = 25), mesoendemic in Mitsamiouli (n = 49), meso- to hyperendemic in Mbeni (n = 77)

### Genotyping of SNPs

In this study, 50 of 151 (33%) samples were genotyped for 24 SNP markers. Among these, 42 samples (1 from Moroni, 17 from Mitsamiouli, and 24 from Mbeni) and 21 SNPs yielded interpretable results (Additional file 1: Table S1). These 42 samples were used for analysis by SNPs. Considering the similar level of genetic diversity included in this sub-sample (He = 0.82) and in all 151 samples (He = 0.83) based on *msp* genes, the representativeness of our sub-sample was confirmed.

Overall, 36 of 42 (85.7%) different genotypes were obtained from 42 samples. Six of 42 (14.3%) isolates grouped into two clusters, defined as a group of parasites with identical SNP barcode: one cluster consisting of 2/42 (4.8%) parasite populations from Mitsamiouli (defined by the barcode TACCXCGACXTAATAAGAXGG; “X” denotes an absence of amplification) and another cluster consisting of 4/42 (9.5%) samples from Mbeni (defined by the barcode TATTCCGTTGCCACTCGATTG). Moreover, among 36 genetically unique isolates, several had a strong linkage since they only differed from each other by a few nucleotides (Additional file 2: Figure S1).

To investigate whether there is an association between barcode and *msp1* and/or *msp*2 (clonality), SNPs (clusters and unique barcodes) were classified as monoclonal or polyclonal. Analysis showed that all clusters were polyclonal. Among the parasites with unique barcodes, 7 of 36 (19.4%) were monoclonal, and 29 of 36 (80.6%) were polyclonal.

### Allelic frequency

Data on allelic frequency showed that several nucleotides predominated at the study sites. At P2 position, adenine (A) number predominated at all sites. At P5 position, cytosine (C) number predominated. At positions P21 and P24, adenine (A) and guanine (G) numbers were seen in the majority of isolates collected in Mitsamiouli and Moroni, respectively. The lowest minority allelic frequency (MAF) was found in position 20 in 5 of 42 (11.9%) isolates calculated from guanine number (Additional file 1: Table S1).

### Genetic diversity

Genetic diversity (He) was estimated from *msp* and SNPs. For *msp* genes, the highest mean diversity was observed in Moroni and Mitsamiouli (He = 0.84 in both sites) (Table 3). There was no significant difference between Moroni and Mbeni (p = 0.48). The mean genetic diversity for all sites was 0.83 (Table 3). For SNPs, He was determined at Mitsamiouli and Mbeni**.** The highest genetic diversity was found at Mitsamiouli (He = 1) and the lowest at Mbeni (He = 0.41).

**Table 3:** Genetic diversity of *P. falciparum* at 3 sites in Grande Comore

Genetic marker	Moroni		Mitsamiouli		Mbeni		All sites	
	N	He	N	He	N	He	N	He
*msp1*	35	0.86	74	0.82	102	0.81	211	0.84
*msp2*	31	0.83	67	0.85	101	0.76	199	0.84
*msp1* + *msp2*	66	0.84	141	0.84	203	0.79	410	0.83

He is the expected heterozygosity, a measure of genetic diversity based on *msp* genotyping, and N is the number of genetically different parasite populations

In addition, He values based on *msp* and SNP were compared in both sites using Fisher's exact test. The He index based on *msp* gene was higher than the index based on SNP in Mbeni (p < 0.01), but an opposite but insignificant trend was observed in Mitsamouili (p = 0.08). Comparison in the whole populations did not show any difference (p = 0.9) (Table 4).

**Table 4:** Comparison of genetic diversity using *msp* and SNPs

Genotyping markers	Mitsamiouli	Mbeni	Both sites
	He (n = 17)	He^*^ (n = 24)	He (n = 24)
*msp*1 + *msp*2	0.79	0.79	0.82
SNPs	1	0.41	0.61

He: heterozygosity. N: the number of isolates

^*^P-value was < 0.05 using Fisher's exact test

## Discussion

The Union of Comoros has entered the phase of malaria elimination after the implementation of several strategies, such as the massive use of insecticide-impregnated bed nets (ITNs), artemisinin-based combination therapy (ACT) for confirmed malaria cases, intermittent preventive treatment in pregnant women (IPTp), and mass drug administration (MDA) with artesunate-mefloquine and primaquine. However, the impact of this intervention on genetic diversity in *P. falciparum* has so far received limited attention and needs to be further investigated. In Grande Comore, genotyping of *msp1* and *msp2* genes revealed a high allelic polymorphism (He = 0.83), comparable to areas of high malaria transmission in Africa [27–29]. For 12 alleles in the *msp1* gene, the K1 family was dominant (55%). For 9 alleles in the *msp2* gene, the FC27 family was most represented (47.4%). These results are similar to those obtained in Madagascar [30] and in Grande Comore in isolates collected in 2013–2016 [17]. Interestingly, the results of the present study differ from those observed in Grande Comore in isolates collected in 2006–2007, in which RO33 family was predominant [17].

Fragment analysis of *msp1* and *msp2* allelic families using agarose gel electrophoresis used in the present study has limitations due to difficulties in defining specific alleles with accuracy for each isolate. To circumvent this drawback, 24 SNPs were used to determine the genetic diversity of *P. falciparum* in Grande Comore for the first time. These 24 SNPs are able to identify different parasites with a high minor allelic frequency [21]. After SNP genotyping, it was found that 85% of isolates are genetically unique. These results suggest that there is a high level of genetic diversity of *P. falciparum* on the island (He = 0.61). The results of SNP genotyping confirm those of *msp1* and *msp2* markers (He = 0.82, p = 0.9).

The comparison of results from the studies concerning *msp* and barcode showed that all clusters were polyclonal. Similarly, among isolates with unique barcodes, the majority (78.9%) were polyclonal as well although data obtained in the present study suggested that the barcode of 21 SNPs cannot predict mixed infections. MOI based on *msp* remains the most powerful tool for determining the number of distinct *P. falciparum* populations in an infection. The average MOI found in this study for all sites was 1.36 for *msp1* and 1.05 for *msp2*. These results are similar to those obtained in Grande Comore in isolates collected in 2013–2016, suggesting a decreasing malaria transmission intensity on this island [17]. Moreover, the present study confirmed the decrease in genetic diversity observed by Huang et al*.* (He = 0.95 before intervention; He = 0.84 after intervention against malaria) [17]. However, comparison of the results of this study with those of Rebaudet et al. (He = 0.63 calculated from microsatellite markers) [16] showed discordance in the level of genetic diversity of *P. falciparum*. This may be due to the choice of *msp* markers used, which are biased due to immune pressure.

Moreover, the considerable level of genetic diversity was found in this study despite trends in decreased malaria transmission in Comoros. Indeed, it has been established that a high genetic diversity of *P. falciparum* is more frequent in areas of high transmission [14]. This observation may provide an explanation for malaria persistence in Grand Comore compared to neighboring islands, despite application of strategies to eliminate malaria, such as MDA. This hypothesis is supported by the fact that Grande Comore alone, compared to three other islands in the Union of Comoros, accounted for 99.4% of malaria cases in 2014 [31]. Indeed, in Grande Comore, there are a large number of water cisterns and ablution basins which serve as mosquito breeding sites [2]. Therefore, local vector control interventions, including larval control, may be needed to improve malaria control.

## Conclusion

Clinical isolates of *P. falciparum* showed high genetic diversity in Grande Comore. This might be one of the reasons for the high malaria transmission in this island, in comparison to neighbouring islands, despite the implementation of several malaria elimination strategies. Further studies on genetic diversity are essential to determine the impact of past interventions. Additionally, they may contribute to the adoption of future strategies for the achievement of malaria elimination in the Union of the Comoros.

## Supplementary information

**Additional file 1: Table S1.** Molecular barcodes for sequenced P. falciparum isolates. Reference P. falciparum clones 3D7/unknown origin (airport malaria, The Netherlands), Dd2/Indochina, HB3/Honduras, and Tm90/Thailand were controls used to differentiate parasite genotypes. MAF, minority allelic frequency. N denotes the number of mixed alleles. X denotes missing nucleotide data. The frequency of minority alleles was determined by counting the number of minority allele in each position.

**Additional file 2: Figure S1.** Phylogenetic tree of SNPs. Samples C13 and C34 are from Mitsamiouli and had the same nucleotide sequence. Samples C54, C48, C44, and C41 had the same sequence. They were all collected in Mbeni. Several isolates were strongly linked. For example, C22 and C24 had a single nucleotide difference. Phylogenetic tree based on SNPs data was constructed using MEGA X UPGMA algorithm.

## Data Availability

All data generated or analysed during this study are included in this article and are available from the corresponding author.

## Abbreviations

ACT: Artemisinin-based combination therapy
HRM: High-resolution melting
IPTp: Intermittent preventive treatment in pregnant women
ITNs: Insecticide-impregnated bed nets
MDA: Mass drug administration
MOI: Multiplicity of infection
*msp*1: Merozoite surface protein-1
*msp*2: Merozoite surface protein-2
RDT: Rapid diagnostic test
SNP: Single nucleotide polymorphisms
UPGMA: Unweighted pair group method with arithmetic mean
