# Supplementary figures and images for: Genetic diversity of Plasmodium falciparum in Grande Comore Island
Source: Malar J. 2020 Sep 3;19:320. doi: 10.1186/s12936-020-03384-5 (PMC7469287; doi:10.1186/s12936-020-03384-5)

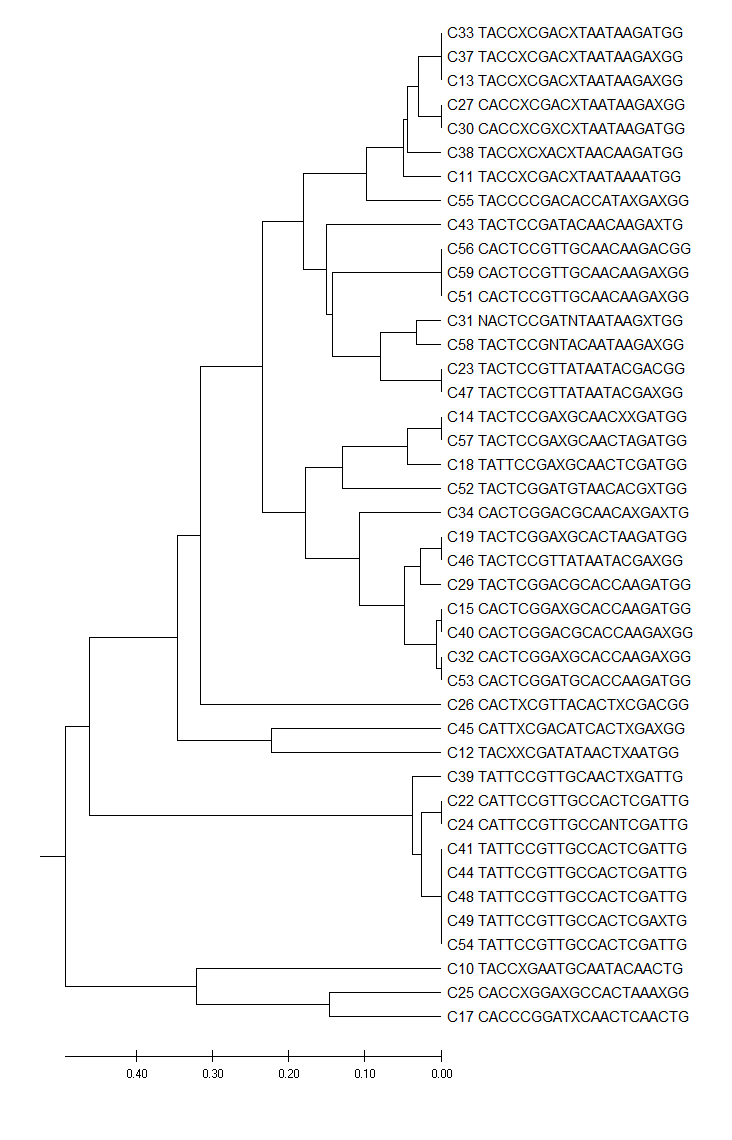

Supplement: Supplementary file 2 — Additional file 2: Figure S1. Phylogenetic tree of SNPs. Samples C13 and C34 are from Mitsamiouli and had the same nucleotide sequence. Samples C54, C48, C44, and C41 had the same sequence. They were all collected in Mbeni. Several isolates were strongly linked. For example, C22 and C24 had a single nucleotide difference. Phylogenetic tree based on SNPs data was constructed using MEGA X UPGMA algorithm. [file 12936_2020_3384_MOESM2_ESM.png]
